# Changes in autophagy, proteasome activity and metabolism to determine a specific signature for acute and chronic senescent mesenchymal stromal cells

**DOI:** 10.18632/oncotarget.6277

**Published:** 2015-11-02

**Authors:** Stefania Capasso, Nicola Alessio, Tiziana Squillaro, Giovanni Di Bernardo, Mariarosa A. Melone, Marilena Cipollaro, Gianfranco Peluso, Umberto Galderisi

**Affiliations:** ^1^ Sbarro Institute for Cancer Research and Molecular Medicine, Center for Biotechnology, Temple University, Philadelphia, PA, USA; ^2^ Department of Experimental Medicine, Biotechnology and Molecular Biology Section, Second University of Naples, Naples, Italy; ^3^ Institute of Bioscience and Bioresources, CNR, Naples, Italy; ^4^ Department of Clinical and Experimental Medicine and Surgery, Division of Neurology, Second University of Naples, Naples, Italy

**Keywords:** mesenchymal stem cells, senescence, metabolism, autophagy, proteasome, Gerotarget

## Abstract

A sharp definition of what a senescent cell is still lacking since we do not have in depth understanding of mechanisms that induce cellular senescence. In addition, senescent cells are heterogeneous, in that not all of them express the same genes and present the same phenotype. To further clarify the classification of senescent cells, hints may be derived by the study of cellular metabolism, autophagy and proteasome activity. In this scenario, we decided to study these biological features in senescence of Mesenchymal Stromal Cells (MSC). These cells contain a subpopulation of stem cells that are able to differentiate in mesodermal derivatives (adipocytes, chondrocytes, osteocytes). In addition, they can also contribute to the homeostatic maintenance of many organs, hence, their senescence could be very deleterious for human body functions.

We induced MSC senescence by oxidative stress, doxorubicin treatment, X-ray irradiation and replicative exhaustion. The first three are considered inducers of acute senescence while extensive proliferation triggers replicative senescence also named as chronic senescence. In all conditions, but replicative and high IR dose senescence, we detected a reduction of the autophagic flux, while proteasome activity was impaired in peroxide-treated and irradiated cells. Differences were observed also in metabolic status. In general, all senescent cells evidenced metabolic inflexibility and prefer to use glucose as energy fuel. Irradiated cells with low dose of X-ray and replicative senescent cells show a residual capacity to use fatty acids and glutamine as alternative fuels, respectively. Our study may be useful to discriminate among different senescent phenotypes.

## INTRODUCTION

Cells may respond to endogenous and exogenous stresses by undergoing senescence, a phenomenon through which the capacity of cell division, growth, and function is lost. There are several genomic stressor events, such as the shortening of chromosome telomeres; non-telomeric DNA damage; excessive mitogenic signals; non-genotoxic stress (i.e. perturbations to chromatin organization). Senescence is considered a barrier against cancer since it blocks the proliferation of transformed cells. On the other hand, cellular senescence contributes to organismal aging. Recently, senescence has been proposed to support other biological processes such as development and tissue repair [1, 2].

In depth understanding of mechanisms that induce cellular senescence is still lacking since we do not have a clear-cut definition of what a senescent cell is. Several features are used to identify senescent cells, such as enlarged and flattened morphology, senescence-associated β-galactosidase activity, senescence-associated heterochromatin foci, altered gene expression, telomere-dysfunction-induced foci, DNA segments with chromatin alterations reinforcing senescence (DNA-Scars), senescence-associated secretory phenotype (SASP). Nevertheless, many of these markers are not senescent cell specific. In addition, senescent cells are heterogeneous in that, not all senescent cells express the same genes and present the same phenotype [1, 3]. Recently, some findings shed a new light on what senescence is trying to reconcile, conflicting data and definitions.

Senescence has to be considered as a dynamic process induced by genetic and epigenetic changes. The early senescence is the turning point, from a transient to a stable cell-cycle arrest that is sustained by p16Ink4a and/or p53-p21 pathways. The progression to full senescence is associated with extensive chromatin remodeling and production of a SASP [2]. In addition, senescent cells should be divided in two classes: acute and chronic senescent cells. Acute senescence is induced by extrinsic stressors, which target a specific population of cells in the tissue and aim to arrest the growth of damaged cells or to be part of physiological phenomena, such as tissue repair and embryonic development. Chronic senescence is induced by prolonged periods of cellular stress, such as continuous proliferation that is associated with DNA replication and consequent accumulation of genomic damages (replicative senescence) [2].

To further clarify the classification of senescent cells, hints may be derived by the study of cellular metabolism, autophagy and proteasome activity in senescence. In this scenario, we decided to study these aspects of senescence in Mesenchymal Stromal Cells (MSC). These cells contain a subpopulation of stem cells that are able to differentiate in mesodermal derivatives (adipocytes, chondrocytes, osteocytes). In addition, they can also contribute to the homeostatic maintenance of many organs [4, 5]. Moreover, MSC are under scrutiny in cellular therapy aiming at treatment of several human diseases.

The growing attention towards stem cell metabolism focused on metabolic needs for maintaining stemness or on metabolic switch that are associated with fate specification and differentiation [6-9]. Less attention has been devoted to analyze the changes in metabolism that are related to senescence of stem cells. This latter issue is of great importance since exhaustion of stem cell compartments contributes to decrements in tissue renewal and function. Metabolism of senescent cells has been investigated mainly in differentiated fibroblasts undergoing oncogenic induced senescence. Some findings showed that senescent cells have increased mitochondrial oxidative metabolism with a key causative role for increased activity of the pyruvate dehydrogenase(PDH) enzyme linking glycolysis and tricarboxylic(TCA) cycle. Others reported a decrease of glucose uptake and metabolism [3, 10]. In addition, other researches evidenced that senescent fibroblasts presented a decline in lipid biosynthesis and an increased fatty acid oxidation. These apparently contradictory results may be reconciled by considering that senescence is cell type, species and context dependent phenomenon. Further complexity arises from the consideration that different genotoxic stressors induce phenotypically different cellular senescent states, which have both common and specific features, mainly at level of expressed genes and secreted factors. Furthermore, the acquisition of a senescent phenotype is a progressive process that, in its initial step, may be reversible before the passage from a transient to a stable cell-cycle arrest [1, 2].

In autophagy process, the lysosomal degradation of cellular molecules and components is a cell response to stress in order to maintain metabolism, and promote cellular viability and fitness [11]. In this scenario, senescence and autophagy represents a way to protect the cell from external and internal stressors. Definitive studies on the relationship between autophagy and senescence are still lacking, since some authors suggest a direct connection between autophagy and senescence and others indicative of an inverse relationship. Indeed, autophagy may promote or counteract senescence depending on cellular context and stress stimuli [11-13]. It appears of great interest to evaluate the role of autophagy in acute and chronic senescence to further clarify commonality and differences among the different types of senescence.

In addition to lysosomes, degradation of damaged proteins (oxidized, misfolded, denatured) occur into proteasome, which is an intracellular protease complex. It is well known that senescence results from the accumulation of deleterious changes over time, this may also be due to reduced activities of proteasomes into cells. Indeed, oxidized and cross-linked proteins tend to accumulate in senescent cells [6, 8, 10]. It remains to be determined, if a declining proteolytic activity of the proteasome may play a role in the several forms of senescence.

## RESULTS

Initially, we evaluated the induction of senescence following treatment with different stressors. The percentage of senescent cells increased almost two times in all conditions but low ionizing radiations (IR) treated cells, which showed a lower increase in senescence (Figure 2). Following induction of senescence we analyzed metabolism, autophagy and proteasome activity. We named D and H, the doxorubicin- and peroxide-treated MSC. Cells treated with low (40 mGy) and high (2000 mGy) radiations were indicated as IRL and IRH, respectively. Replicative senescent MSC and control cultures were named Rep and CTRL, respectively.

**Figure 1:**
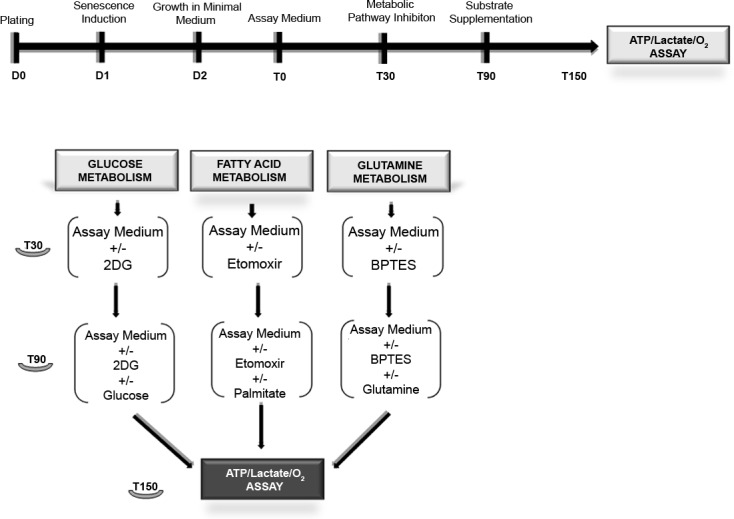
Workflow for metabolic assays MSC were in growth medium (D0). After 24h stress-induced senescence was triggered as described in methods (D1). Chronic senescent cells were plated in growth medium (D0). After 24 hours, we performed medium change without use of stressors (D1). The day after (D2), we replaced the growth medium with the substrate-limited medium. After 24 hours (T0), we changed the substrate-limited medium with the assay medium. After 30 min (T30) in a group of samples, we added the metabolic pathway inhibitor drugs (2D-glucose, etomoxir and BPTES). After 60 min (T90), we added the relative substrates (glucose, palmitate and glutamine) in all samples. Following 60 min incubation (T150), we performed the lactate, ATP and O_2_ assays.

**Figure 2:**
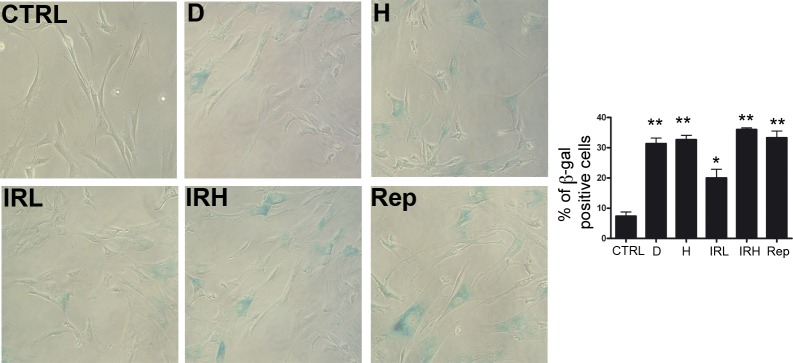
Induction of senescence in MSC cultures Representative microscopic fields of acid beta-galactosidase (blue) in treated and control cells are shown. The graph shows mean percentage value of senescent cells (± SD, *n* = 3, *p < 0.05).

### Senescent cells evidenced metabolic inflexibility

In order to avoid cytotoxic effect of metabolic inhibitors (etomoxir, 2-DG and BPTES), we carried out preliminary experiments to determine the effective drug concentrations, which did not induce cell death, as evaluated by trypan bleu dye exclusion test (data not shown).

**Figure 3:**
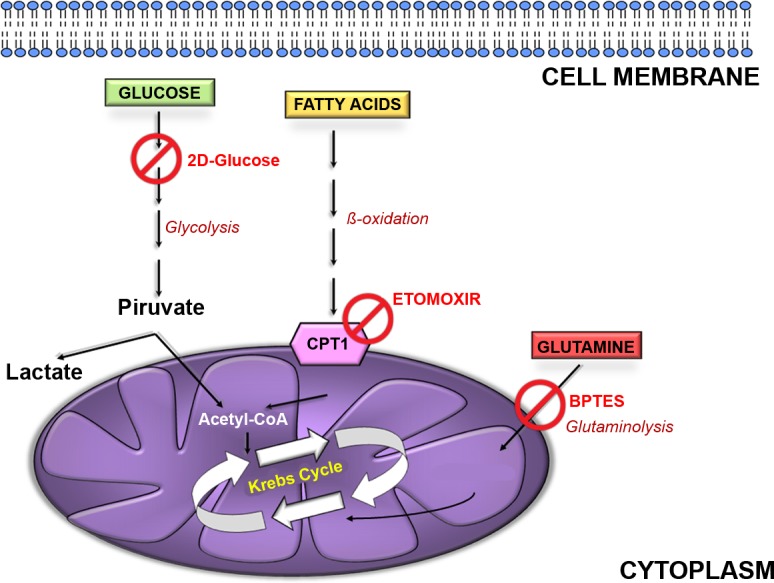
Cellular energetic pathways Glucose, fatty acids and amino acids (glutamine in the example) are the main energy fuels for the cells. Glucose is metabolized to pyruvate through glycolysis into cytoplasm. This can be converted to lactate or enter mitochondria to be converted in acetyl-CoA. This is combined with oxaloacetate to give citrate and undergo further oxidation in trycarboxylic acid (TCA) cycle. Fatty acids can enter mitochondria as acyl-CoA by mean of carnitinepalmitoyltransferase 1 (CPT1) and here can be oxidized to acetyl-CoA and fuel the TCA cycle. Glutamine can be converted to glutamate by glutaminase (GLS) and further metabolized to α-ketoglutarate and then oxaloacetate to fuel TCA cycle. 2-deoxyglucose inhibits glycolysis; etomoxir impair acyl-CoA transfer into mitochondria; BPTES inhibits GLS activity.

Healthy cells can oxidize several substrates for energy production and can adapt nutrient oxidation to nutrient availability (Figure 3). This last property is called metabolic flexibility. A “metabolic flexible” individual switches freely among the principal nutrients (carbohydrates, lipids and amino acids) depending on environmental cues and physiological needs [16]. This capacity is lost in the elderly; in fact, aging is associated with metabolic dysfunction [17, 18]. For this reason, we aimed to evaluate to what extent metabolic inflexibility was present in senescent cells. We grew senescent MSC in media supplemented with a single nutrient (glucose, or palmitate or glutamine) and evaluated ATP levels, basal oxygen consumption, oxygen employed for mitochondrial production and maximal respiration.

**Figure 4:**
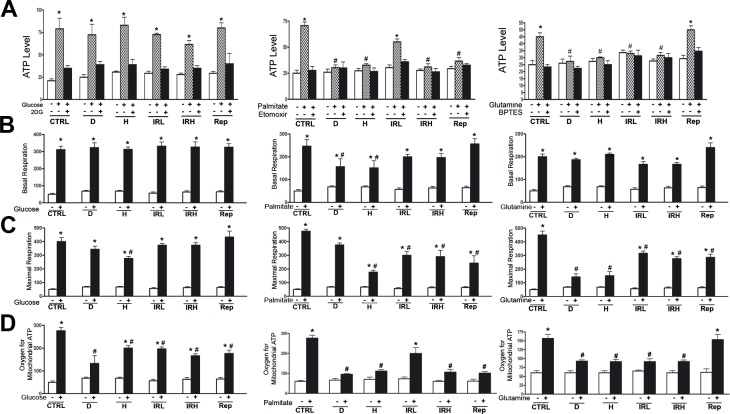
Evaluation of metabolic activity in senescent cells **A.** *ATP level measurement.* Intracellular ATP level was determined using the ATP Colorimetric/Fluorometric Assay. Cells were incubated in medium containing glucose (left graph) or palmitate (middle graph) or glutamine (right graph). The ATP level was measured in absence of energy substrate (white bars), in presence of specific substrate (gray bars) and in the presence of substrate and its inhibitor (black bar). For every condition, the asterisk (*) denotes significant differences (*p* < 0.05) between ATP level in a medium with a specific substrate (gray bar) and a medium without substrate plus its inhibitor (black bar). Data are expressed in arbitrary units (± SD, *n* = 3). **B.** *Basal oxygen consumption rate.* We used MitoXpress^®^ Xtra assay for measurement of extracellular oxygen consumption rates (OCR). Cells were incubated in medium containing glucose (left graph) or palmitate (middle graph) or glutamine (right graph). For every condition, the asterisk (*) denotes significant differences (p < 0.05) between OCR in a medium with a specific substrate (black bar) and a medium without energy substrates (white bar). The hash symbol (#) denotes differences between ATP in control healthy cells and senescent cells (D, H, IRL, IRH, Rep). Data are expressed in arbitrary units (± SD, *n* = 3). **C.** *Maximal respiration rate.* OCR was measured following sequential addition of FCCP, that is, a protonophore and uncoupler of oxidative phosphorylation in mitochondria. Cells were incubated in medium containing glucose (left graph) or palmitate (middle graph) or glutamine (right graph). For every condition the asterisk (*) denotes significant differences (*p* < 0.05) between maximal OCR in a medium with a specific substrate (black bar) and a medium without energy substrates (white bar). The hash symbol (#) denotes differences between OCR in control healthy cells and senescent cells (D, H, IRL, IRH, Rep). Data are expressed in arbitrary units (± SD, *n* = 3). **C.** *Oxygen for mitochondrial ATP production.* OCR was measured following sequential addition of olygomycin that is inhibitor of the F_0_ part of H+/ATP-synthase in mitochondria. Cells were incubated in medium containing glucose (left graph) or palmitate (middle graph) or glutamine (right graph). For every condition the asterisk (*) denotes significant differences (*p* < 0.05) between OCR in a medium with a specific substrate (black bar) and a medium without energy substrates (white bar). The hash symbol (#) denotes differences between OCR in control healthy cells and senescent cells (D, H, IRL, IRH, Rep). Data are expressed in arbitrary units (± SD, *n* = 3).

Healthy MSC appeared metabolic flexible (Figure 4A, 4B, 4C, 4D). Indeed, their ATP levels increased when cells were incubated in medium containing only glucose, or palmitate or glutamine compared with medium devoid of nutrient. The ATP increase was nutrient-specific, since it was negatively affected by the presence of metabolic inhibitors (Figure 4A).

Senescent cells were less flexible. All senescent MSC were able to use glucose for ATP production, but only those treated with low dose of radiation (IRL) evidenced a residual ability to increase ATP levels in the presence of palmitate (Figure 4A middle graph). In addition, only Rep cells were able to use glutamine as fuel for ATP production (Figure 4A right graph).

The oxygen consumption assay allowed us to determine basal oxygen consumption, the oxygen employed for mitochondrial ATP production and maximal respiration. In the presence of glucose, all senescent cells were able to produce mitochondrial ATP with a significantly impairment in production compared with control cells (Figure 4D left graph). In media containing palmitate only in IRL the oxygen for mitochondrial ATP production is not significantly reduced compared with healthy cells, while other senescent cells showed residual ability to produce mitochondrial ATP in presence of palmitate (Figure 4D middle graph). Only Rep cells used glutamine as fuel for mitochondrial ATP biosynthesis in a manner that was almost equivalent to healthy cells (Figure 4D right graph). Globally, all these data are in agreement with those we obtained with assay measuring the total ATP cellular levels.

Healthy cells can respond to sudden changes in ATP demand mainly by energetic capacity of mitochondria. The measurement of maximal respiratory capacity (oxygen consumption in the presence of oxidative phosphorylation uncoupler, such as FCCP) reflects how cells react to an increased ATP demand. In media containing glucose, all types of senescent cells, but H, showed the ability to cope with increase in ATP requirements (Figure 4C left graph). This capacity was not evidenced in media containing palmitate or glutamine as energy fuels (Figure 4C middle and right graphs).

In media containing glucose or glutamine, the basal oxygen consumption of senescent cells was not reduced compared to healthy cells (Figure 4B left and right graphs). In media with palmitate, the basal respiration was reduced in senescent cells with the exception of IRL, IRH and Rep (Figure 4B middle graph).

### Lactate

Proliferating stem cells rely mainly on the first part of the energy production process (glycolysis) to generate ATP rapidly in the cytoplasm and to reduce the cytoplasmic NAD+/NADH ratio. Great part of the resulting pyruvate is transformed in lactate by the lactate dehydrogenase (LDH), which reconverts NADH in NAD+. The NAD+ allows glycolysis to persist, and the lactate is secreted from the cell [19]. As expected, healthy MSC cultures showed the ability to produce lactate suggesting that they can use aereobic glycolysis for energy production (Table 1). This ability is almost completely lost in all forms of senescent cells (Table 1).

**Table 1:** Lactate level measurement Lactate level was determined using the fluorimetric lactate Assay Kit

	CTRL	D	H	IRL	IRH	Rep
Glucose	65.0±9.7	20.5±2.2 #	23.9±3.1 #	15.9±2.7 #	15.0±2.2 #	20.1±2.2 #
Glucose 2DG	35.4±4.6 *	18.7±2.4	15.7±1.8	10.5±1.7	13.2±1.8	13.4±1.7

Cells were incubated in medium containing glucose with and without 2D-glucose, that is an inhibitor of glycolysis flux.

The hash symbol

(#) denotes differences between lactate level in control healthy cells and senescent cells (D, H, IRL, IRH, Rep).

For every condition the asterisk

(*) denotes significant differences (*p* < 0.05) between lactate level in media containing glucose with and without 2D-glucose.

Data are expressed in arbitrary units (± SD, n = 3).

### Autophagy was maximally impaired in some senescent phenotypes

**Figure 5:**
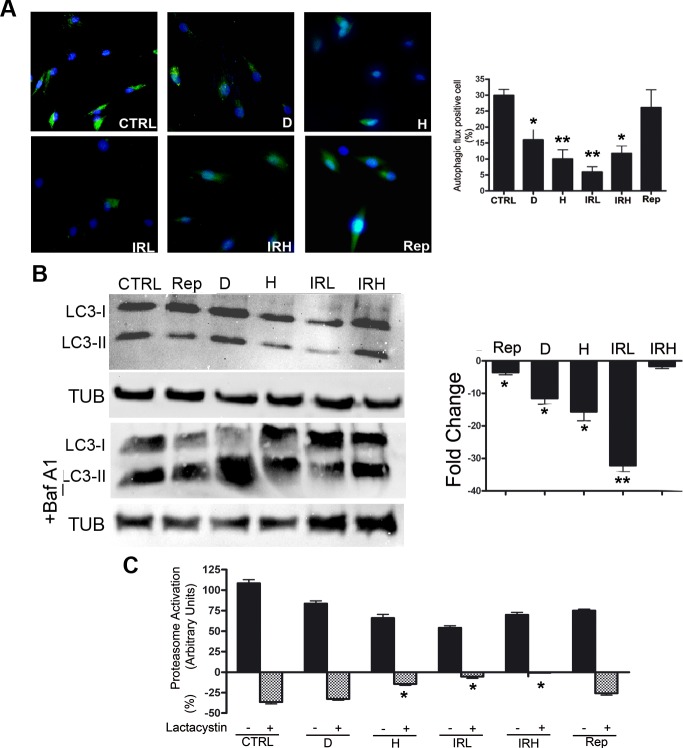
Autophagy and proteasome activity assays **A.** *Cyto-ID assay*. Representative microscopic fields of cells with active autophagy (green) are shown. Nuclei were counterstained with Hoechst 33342 (blue). The graph shows mean percentage value of Cyto-ID-positive cells (± SD, *n* = 3, *p < 0.05, **p < 0.01). **B.** *Autophagic flux evaluation* – The picture shows western blot detection of LC3-I and LC3-II in senescent and control MSC. Following induction of senescence with different stressors cells were incubated for six hours and then harvested for western blot analysis. Two hours before the end of cell sample preparation, senescent and control MSC cultures were incubated with 100 nMB afilomycin A1 (inhibitor of lysosomal degradation) or PBS to detect autophagic flux. We used Gel Doc 2000 Gel Documentation Systems (Bio-Rad, CA, USA) to measure LC3-I and II band intensities that were normalized with tubulin (TUB). We determined autophagic flux (AF) for LC3 II as follows: IR-treated MSC AF = (IR-treated MSC + Bafylomycin A1) - (IR treated MSC + PBS); Control MSCs AF = (Control MSC + Bafylomycin A1) - (Control MSC + PBS). Change in autophagic flux (ΔAF) between IR-treated and control MSC was calculated as ΔAF = IR-treated MSC AF - Control MSC AF. The graph shows AF changes in IR-treated MSC compared to control cultures. Data are expressed in change folds (*n* = 3; *p < 0.05, **p < 0.01). **C.** *Proteasome activity.* Following induction of senescence with different stressors, cells were incubated for six hours and then harvested for fluorimetric assay determination of proteasome activity. Two hours before the end of cell sample preparation, senescent and control MSC cultures were incubated with 25 μM lactacystin (a specific and potent inhibitor of proteasomes) or PBS. The graph shows proteasome activity in control and senescent cells both in the presence and absence of lactacystin. Data are expressed in arbitrary units (*n* = 3; *p < 0.05).

We measured by western blot the levels of LC3-I and LC3-II that are two isoforms of the microtubule-associated protein 1 light chain 3 (LC3), a reliable marker of autophagosome. We determined the autophagic flux by tracking the conversion of LC3-I proteins to LC3-II that occurs when autophagy is induced [20]. We determined the level of LC3 isoforms, both in the presence and absence of bafilomycin A1, an inhibitor of lysosomial degradation. In D, H and IRL cells we observed a reduction of autophagic flux, suggesting an impairment of the autophagy process (Figure 5B). Inhibition of autophagy in these cells was confirmed by Cyto-ID Autophagy Detection assay. The percentage of cells expressing active autophagic vacuoles was significantly decreased in D, H and IRL cells (Figure 5A). We detected also a reduction of cells with active vacuole in IRH sample. This may suggest that process of autophagy in these was not blocked (flux is comparable to control) but the number of active vacuoles is reduced.

### Proteasome activity was reduced only in some type of senescent cells

We used a 20S proteasome assay to evaluate the activity of the ubiquitin-proteasome pathway. This is the main proteolytic system in cells, which catalyzes the selective degradation proteins with abnormal conformation and of those with short half-life (regulatory proteins) [21]. We measured chymotrypsin-like peptidase activity both in the presence and absence of lactacystin, a specific and potent inhibitor of proteasomes [22]. If in a given experimental condition, the proteasome activity is no further reduced by the presence of protease inhibitor, this indicates a reduction of 20S proteasome functionality. The IRL, IRH and H senescent cells showed a significant impairment of 20S proteasome activity compared with control (Figure 5C).

## DISCUSSION

Cellular senescence is involved in several biological processes such as tumor suppression, tumor promotion, tissue repair, development and aging. How possible is its involvement in apparently contrasting phenomena? The answer resides in the consideration that, there exist different types of senescent cells, which possess a “common background” of shared properties and specific features. Dissecting molecular pathways underlying the multi-step progression of senescence and the onset of acute *versus* chronic senescent cells may trigger the discovery of new therapies for senescence-related diseases and aging [2, 3]. In this scenario, the analysis of metabolic needs and of the processes involved in clearance of exhausted cellular organelles and molecules may allow for the identification of commonalities and differences among the various senescent phenotypes, given the relationship of these phenomena with trigger and sustainment of senescence.

We decided to analyze the senescence in MSC, since to our knowledge there are only a few papers that partially address this topic in spite of the key role of MSC in hematopoiesis and in the homeostatic maintenance of many organs and tissues.

### MSC senescent cells produce ATP *via* oxidative phosphorylation and are metabolically inflexible

Partial utilization of glucose through anaerobic glycolysis and shunt of its intermediates, through the pentose phosphate pathway provides a sufficient production of ATP, reducing cofactors and substrates to meet anabolic requirements of stem cells for proliferation and self-renewal. As stem cells differentiate, they change their metabolic needs, since progenitors and differentiated cells depend on large amounts of energy to sustain homeostasis and increasingly specialized functions. This is accomplished through complete glucose oxidation within TCA cycle [23]. Our data are in good agreement with these premises since in healthy cultures of MSC, which contain different cell populations, we evidenced ATP production either through TCA cycle or anaerobic glycolysis (Figure 4). This latter event should occur mainly in the MSC stem cell population, while mitochondrial ATP production mainly in committed and differentiated cells. Of interest, cells in healthy cultures had the capacity to freely switch between alternative fuels (sugars, fats and amino acids) as it occurs in physiological conditions (metabolic flexibility).

All types of senescent MSC cultures did not rely on anaerobic glycolysis since lactate production was almost completely abolished (Table 1). This may suggest that following induction of senescence in MSC cultures the presence of stem cells is lost or significantly reduced. Senescent MSC cultures appeared to produce ATP mainly *via* oxidative phosphorylation. Nevertheless, they lost the capacity to freely utilize different energy sources and relied mainly on glucose as energy fuel. IRL and Rep senescent cells showed a residual capacity to use fatty acid and glutamine as alternative fuels, respectively. The fact that different senescent inducers promote the use of different metabolic pathways may be used to discriminate between senescent phenotypes. In addition, it should be underlined that the metabolic inflexibility we detected in senescent cells is a further link with aging and related diseases [16, 24].

### Autophagy flux is impaired in some senescent forms

In some experimental models, senescence onset is dependent on a preliminary autophagy induction. In contrast, in other contexts the inhibition of autophagy promotes senescence. We evidenced that in all types of MSC acute senescence, but IRH, the autophagy flux is heavily impaired suggesting the autophagy counteracts deteriorative processes, and its decline triggers senescence. This did not occur in replicative senescence. It remains to be determined if most of the acute stressors that induce MSC senescence are associated with a decline in autophagic functions. Indeed, in human fibroblasts autophagy is activated during acute senescence induced by oncogene activation [25]. To reconcile these opposite events, we may speculate that cells try to contend with stress by activating autophagy that eliminates damaged components. In this context, autophagy protects from senescence and impairment of its function may promote senescence. On the other hand, if autophagy cannot counteract stress-induced damage, it may induce senescence.

### Proteasome activity is reduced in specific senescent phenotypes

The proteasome complex contains a threonine protease that degrades intracellular proteins, which are misfolded, denatured, or otherwise damaged. It can also eliminate healthy proteins for normal cellular turn-over. It is reasonable to hypothesize that accumulation of damaged proteins may undermine normal cellular functions and induce senescence. Indeed, proteasome activity is impaired in some forms of senescence: either acute senescence in U937 leukemic cells or chronic replicative senescence in human fibroblasts [6, 8].

In our model, we detected a reduction of proteasome activity in H, IRL and IRH cells. This decrease only in specific forms of senescence may be related to the high production of reactive oxygen species as it occurs in cells treated with peroxide hydrogen or in irradiated cells. This may affect proteasome functions. Indeed, mild or transient oxidative stress up-regulates positively proteasome activity in cells, while severe or sustained oxidative stress impairs the function of the proteasome activity [26].

### Is it possible to create a data analysis algorithm that could effectively identify different forms of senescence?

The differences in metabolic needs and in the activity of proteasome and autophagic vacuoles among the several forms of senescent MSC we analyzed allow identification of a specific algorithm for every phenotype (Table 2). For example, Rep MSC can use glucose and partially glutamine as fuels. Moreover, they did not show change in proteasome and autophagy functions. On the contrary, IRL, MSC use glucose and fatty acids for their energetic needs and showed impaired activity of proteasome and autophagy. It should be underlined that senescence is cell type, species and context dependent phenomenon. For this reason, the identified algorithm holds the attention of researchers and physicians interested in understanding the physiological role of MSC in tissue homeostasis and regeneration and for those aiming to use MSC in cell therapy. Our research suggests that senescent cells in a batch of MSC that will be delivered to patients may affect their therapeutic potential. Thus, it is essential to evaluate the percentage of senescent cells in each batch of MSCs that will be delivered to patients.

**Table 2:** Data analysis algorithm to identify different senescent phenotypes For every form of senescence (Rep, D, H, IRL, IRH) are indicated the negative changes in proteasome activity and autophagic flux (↓) and the presence of active metabolic pathways

	Proteasome Activity	Autophagic flux	Glucose metabolism	Fatty Acid metabolism	Amino Acid metabolism
Rep			Active		Active
D		↓↓	Active		
H	↓	↓↓	Active		
IRL	↓	↓↓	Active	Active	
IRH	↓		Active		

In conclusion, our finding may pave the way to carry out similar investigation on other cell types and with other stressors; this is to have a complete perspective of senescence.

## MATERIALS AND METHODS

### MSC cultures

Bone marrow was obtained from three healthy donors (age range, 6-10 years old) who had provided informed consent. We separated cells on a Ficoll density gradient (GE Healthcare, Italy), and the mononuclear cell fraction was collected and washed in PBS. We seeded 1-2.5 × 10^5^ cells/cm^2^ in alpha-MEM containing 10% FBS and bFGF. After 72 hours, non-adherent cells were discarded, and adherent cells were cultivated to confluency. Cells were then further propagated for the assays reported below. All cell culture reagents were obtained from Euroclone Life Sciences (Italy).

### Acute and chronic senescent MSC

For induction of acute senescence, we used three different stressors: irradiation, doxorubicin and peroxide hydrogen treatments. Chronic senescent MSC were obtained by extensive *in vitro* cultivation for 30 days (replicative senescence) as we already described [14].

Irradiation treatment: Exponentially growing cells (passage 3) were irradiated with 40 and 2000 mGy X-ray at room temperature. X-rays were administered *via* a Mevatron machine (Siemens Italy) operating at 6 MeV. Following irradiation, cells were cultivated for 48 hours before carrying out further experiments.

Doxorubicin treatment: Cells were incubated with 1 μM doxorubicin in complete culture medium for 24 hours, then medium was discarded and cells were incubated for 24 hours in fresh medium before further analysis.

Peroxide hydrogen treatment: Cells were incubated with 300 μM H_2_O_2_ for 30 minutes in complete medium, then medium was discarded and cells were incubated for 48 hours in fresh medium before further analysis.

### *In situ* senescence-associated beta-galactosidase assay

The percentage of senescent cells was calculated by the number of blue, beta-galactosidase-positive cells out of at least 500 cells in different microscope fields, as already reported [15].

### Workflow forO_2_, ATP and lactate assays

The complete workflow for the metabolic assays is depicted in Figure 1. We seeded 1×10^6^ cells in 150-mm dishes with alpha-MEM containing 10% fetal bovine serum, 2 ng/ml bFGF, 2 mM L-glutamine, 100 U/ml penicillin, and 100μg/ml streptomycin (D0 -growth medium). Afterwards, 24 hour stress-induced senescence was triggered as described above (D1). Chronic senescent cells were plated in growth medium (D0). After 24 hours, we performed medium change without the use of stressors (D1).

The day after (D2), we replaced the growth medium with the substrate-limited medium containing DMEM (code A14430 from GIBCO LifeTech Italy), 1% FBS 0.5mM glucose, 1mM glutamax, 0.5mM carnitine. Following 24 hour incubation (T0), we replaced the substrate-limited medium with the assay medium (PBS containing MgCl_2_ and CaCl_2_, 5mM HEPES, 2.5mM glucose, 0.5mM carnitine); after 30 min (T30) in a group of samples, we added the metabolic pathway inhibitor drugs (48mM 2D-glucose, 0.25 mMetomoxir and 3 μM BPTES). After 60 min (T90) we added the relative substrates(25mM glucose, 200 μM palmitate and 4mM glutamine) in all samples. Following 60 min incubation (T150) we performed the lactate, ATP and O_2_ assays.

### Oxygen consumption assay

We determined the intracellular oxygen levels in several experimental conditions to evaluate carbohydrates, lipids and glutamine contribution to oxygen consumption. MitoXpress^®^ Xtra assay (Luxcel Biosciences, Ireland) allows measurement of extracellular Oxygen Consumption Rates (OCR) using a oxygen-sensing fluorophore that is quenched by O_2_ through molecular collision, thus, the amount of fluorescence signal is inversely proportional to the amount of extracellular O_2_ in the sample. Initially, we measured basal respiration, later, we sequentially added oligomycin and FCCP, which target components of the Electron Transport Chain (ETC) in the mitochondria to reveal key parameters of metabolic function. The two compounds allowed for the determination of oxygen employed for mitochondrial ATP production and maximal respiration, respectively. We determined the OCR according to manufacturer's instructions.

### ATP assay

ATP measurement was determined using the ATP Colorimetric/Fluorometric Assay Kit (BioVision, CA, USA), which takes advantage of glycerol phosphorylation to generate a product that is easily quantified by fluorometric (Ex/Em = 535/587 nm) methods. Measurements were performed with the fluorometric assay in 96-well black bottom plates following the manufacturer's instructions.

### Lactate assay

Lactate measurements were determined using the lactate Assay Kit (Sigma-Aldrich Italy, Italy) by an enzymatic assay, which results in a fluorometric (λex = 535 nm/λem = 587 nm) product, proportional to the lactate concentration. Measurements were performed with the fluorometric assay in 96-well black bottom plates following the manufacturer's instructions.

### Cyto-ID autophagy detection kit

The Cyto-ID^®^ Autophagy Detection Kit (Enzo Life Science, NY, USA) measures autophagic vacuoles and monitors autophagic flux in live cells using a cationic amphiphilic dye that selectively labels autophagic vacuoles. We determined the percentage of Cyto-ID-positive cells according to manufacturer's instructions.

### Western blotting

Cells were lysed in a buffer containing 0.1% Triton for 30 minutes at 4°C. 20 μg of each lysate was electrophoresed in a polyacrylamide gel, and electroblotted onto a nitrocellulose membrane. All the primary antibodies were used according to the manufacturers’ instructions. Immunoreactive signals were detected with a horseradish peroxidase-conjugated secondary antibody (SantaCruz, CA, USA) and reacted with ECL plus reagent (GE Healthcare, Italy).

### Proteasome activity

Proteasome Activity was determined using the 20S proteasome activity assay Kit (Merck-Millipore Italy, Italy) which is a simple method that recognizes the substrate LLVY. The assay is based on detection of the fluorophore 7-Amino-4- methylcoumarin (AMC) after cleavage from the labeled substrate LLVY-AMC. The free AMC fluorescence can be quantified using a 380/460 nm filter set in a fluorometer. Measurements were performed with the fluorometric assay in 96-well black bottom plates following the manufacturer's instructions.

### Statistical analysis

Statistical significance was evaluated using ANOVA analysis followed by Student's t and Bonferroni's tests. We used mixed-model variance analysis for data with continuous outcomes. All data were analyzed with a GraphPad Prism version 5.01 statistical software package (GraphPad, CA, USA).
